# The cost-effectiveness of a proportionate parenting programme for primary caregivers and their child: an economic evaluation using evidence from the E-SEE Trial

**DOI:** 10.1186/s12913-022-08220-x

**Published:** 2022-06-23

**Authors:** Edward Cox, Simon Walker, Sarah Blower, Filipa Sampaio, Tracey Bywater, Gerry Richardson

**Affiliations:** 1 Centre for Health Economics, University of York, York, UK; 2 Department of Health Sciences, University of York, York, UK; 3 Department of Public Health and Caring Sciences, Uppsala University, Uppsala, Sweden

**Keywords:** Cost-effectiveness, Incredible years, RCT, E-SEE Steps, Parenting strategies, Childhood health

## Abstract

**Background:**

Behavioural and mental disorders have become a public health crisis; averting mental ill-health in early years can achieve significant longer-term health benefits and cost savings. This study assesses whether the Enhancing Social-Emotional Health and Wellbeing in the Early Years (E-SEE-Steps)—a proportionate universal delivery model comprising the Incredible Babies book (IY-B) and the Incredible Years Infant (IY-I) and Toddler (IY-T) parenting programmes is cost-effective compared to services as usual (SAU) for the primary caregiver, child and dyad.

**Methods:**

Using UK data for 339 primary caregivers from the E-SEE trial, we conducted a within-trial economic evaluation assessing the cost-effectiveness of E-SEE Steps. Health outcomes were expressed in quality-adjusted life-years (QALY) and costs in UK pounds sterling (2018–19). Missing data were populated via multiple imputation and costs and QALYs discounted at 3.5% per annum. Cost-effectiveness results were conducted for primary caregivers, children and dyad using econometric modelling to control for patient co-variables. Uncertainty was explored through scenario and sensitivity analyses.

**Results:**

The average cost of E-SEE Steps intervention was £458.50 per dyad. E-SEE Steps was associated with modest gains in primary caregiver HRQoL but minor decrements in child HRQoL compared to SAU. For primary caregivers, E-SEE Steps was more effective (0.034 QALYs) and more costly (£446) compared to SAU, with a corresponding incremental cost-effectiveness ratio (ICER) of £13,011 per QALY. In children, E-SEE Steps was strictly dominated with poorer outcomes (-0.005 QALYs) and greater costs (£178) relative to SAU. QALY gains in primary caregivers exceeded those QALY losses found in children, meaning E-SEE Steps was more effective (0.031 QALYs) and costly (£621) for the dyad (ICER: £20,062 per QALY). All scenario analyses found E-SEE Steps cost-effective for the dyad at a £30,000 per QALY threshold. Sensitivity analyses found significant cost reductions from expansions in programme delivery and attendance.

**Conclusions:**

E-SEE Steps achieved modest health gains in primary caregivers but small negative effects on children and was more costly than SAU. E-SEE Steps appears cost-effective for the dyad, but the results should be interpreted with caution given the potential detrimental impact on children.

**Trial registration:**

ISRCTN11079129; Pre participant trial enrolment, 11/05/2015

**Supplementary Information:**

The online version contains supplementary material available at 10.1186/s12913-022-08220-x.

## Background

Mental health problems have become a public health crisis. In the UK mental health problems represent the largest single cause of ill-health and disability, with 1 in 4 people now experiencing a mental health issue each year [1]. The annual economic costs of mental health problems in the UK stands at approximately £118 billion per year, prompting significant policy interest in preventative interventions, particularly those aimed at improving early cognitive and social development, and in combatting intergenerational transmission of mental and behavioural disorders [1–3]. Preventing mental ill-health in early years can alleviate large disease and financial burdens to children, their families and wider society [4, 5].

Ineffective parenting strategies and parental emotional distress are known to impact negatively on child social and emotional wellbeing, while children whose parents have mental health problems are more likely to present with similar problems themselves [6, 7]. Early intervention programmes, targeted at vulnerable child populations, have been shown as effective preventative strategies in a number of settings [8]. Interventions aimed towards improving the home environment, parenting skills, positive parent–child interactions and understanding of child development and safety issues, can act as a more effective, and less costly, alternative to corrective care [9]. Accordingly, UK policy guidelines and recent literature reviews promote interventions which enhance the social and emotional well-being of vulnerable young children, recommending home visits, early education, childcare and parenting programmes [10, 11]. Targeted child services have the potential for longer-term health benefits and cost savings, with positive feedback within and between generations.

The Incredible Babies book (IY-B) is a guide designed to help parents understand and journal a baby’s physical, social, emotional and language development; the Incredible Years (IY) Infant (IY-I) and Toddler (IY-T) parenting programmes are manualised parent education and training interventions which use group-based components and materials to enhance the social and emotional well-being of children aged 0–1 and 1–3 years, respectively [12]. IY programmes have been evaluated in a variety of contexts and shown to confer sizeable benefits, particularly in highly distressed families [13, 14]. The aim of this economic analysis was to consider the cost-effectiveness of E-SEE Steps, a multi-layer intervention combining universal IY-B with targeted IY parenting programmes, using evidence from the E-SEE (Enhancing Social-Emotional Health and Wellbeing in the Early Years) trial.

## Methods

### E-SEE trial

The E-SEE trial was a community-based pragmatic two-arm randomised controlled trial that evaluated the E-SEE Steps programme compared with services as usual (SAU). At baseline, all intervention parents received IY-B and depending on level of need, as indicated by parental depression scores or child social emotional wellbeing (Patient Health Questionnaire ≥ 5; or Ages and Stages Questionnaire: Social and Emotional, 2nd edition ≥ Monitoring Zone), parents were invited to participate in the IY-I programme (10 weeks, 2 h/week) at 2 months post-baseline and/or the IY-T programme (12 weeks, 2 h/week) at 9 months [15, 16]. The trial recruited parents of children aged 0–2 years from predominately disadvantaged areas in England. Further details about the trial are available elsewhere [17–19].

### Resource use and costs

Parental- and child-related resource use data was collected from primary-caregivers at baseline, 2-months, 9-months, and 18-months post-baseline follow-up using a modified service use questionnaire. Baseline forms recorded resource use two-months prior to trial entry.

Resource use and costs were grouped into the following categories: intervention-related; primary-care; secondary-care; mental health care; community service; social service; childcare; and absent workdays. Intervention-related unit costs were based on recorded trial costs; childcare costs were informed by relevant surveys [20, 21]; health care, social care and community service costs were sourced from published UK sources [22, 23]; and absent workday productivity losses were estimated using the human capital approach [24]. Costs reflected UK pounds sterling at 2018–19 prices. Unit costs were inflated where necessary [23]. Unit costs are provided in Appendix file 1 in Supplemental Materials.

Resource use from the E-SEE Steps programme comprised: the IY-B books provided to staff and primary caregivers; the staff, materials and operational arrangements used to train group leaders in the IY-I and IY-T programmes; the staff time and materials used in IY-I and IY-T group-sessions; and the follow-up correspondence provided to primary caregivers between group sessions (i.e. letters, texts, phone calls and home visits). Intervention costs were taken from trial records and were applied to primary caregiver costs. To explore the potential for economies-of-scale, intervention-related costs were divided into variable and fixed costs (Appendix 1).

Total costs were aggregated for each primary caregiver, enrolled child and dyad by multiplying the total number of each resource item utilised over the trial period by their respective unit costs and summating. Costs were estimated from three perspectives: 1) a public health sector perspective (NHS and Personal Social Services); 2) a family perspective considering childcare and absenteeism; and 3) a broader perspective encompassing both public- and family-related costs. The base case cost-effectiveness analysis was conducted from a public health sector perspective. Scenario analyses explored alternative costing perspectives and minimum, maximum, and site-specific intervention costs. Sensitivity analyses explored how changes in group-session attendance and the number of programmes a trained practitioner ultimately delivers impacts average programme costs and cost-effectiveness.

### Outcomes

The primary outcome used in the cost-effectiveness analysis was quality-adjusted life years (QALYs), a composite measure of health encompassing both morbidity and mortality (with one QALY equalling a year in perfect health).

The HRQoL of primary caregivers was estimated using the EQ-5D-5L questionnaire collected at baseline, 2, 9- and 18-months post-randomisation. The EQ-5D-5L is a descriptive system requiring individuals to rate their health in accordance with five levels of severity across five health dimensions (mobility, self-care, usual activity, pain/discomfort and anxiety/depression), resulting in a total of 3,125 possible health states. In line with guidance from the National Institute for Health and Care Excellence (NICE), base case HRQoL weights were calculated using a published mapping of EQ-5D-5L responses onto those HRQoL values reported from the EQ-5D-3L instrument [25, 26]. Preference values based on a study of population values of the EQ-5D-5L were used in a scenario analysis.

The HRQoL of children was informed using the Strengths and Difficulties Questionnaire (SDQ) completed by primary caregivers at 18 months post-randomisation. The SDQ is a behavioural screening questionnaire designed to measure emotional and behavioural problems for children and young people. A published mapping of SDQ responses onto the Child Health Utility (CHU9D) questionnaire, a generic preference-based measure of paediatric HRQoL, was used to derive study HRQoL weights for children [27]. Baseline HRQoL was assumed to be equal to that reported in SAU at 18 months for both arms as collection of baseline values was not feasible.

Adult and child QALYs were estimated separately using an area under the curve approach with linear interpolation between time points [28].

### Analysis

Cost-effectiveness results were calculated over the trial time-horizon (18 months) and were informed using estimates of primary caregiver and child costs and QALYs that control for relevant participant co-variables. Regression analyses controlled for treatment allocation, child age, adult age, child gender, highest qualification, and relationship status. Baseline HRQoL scores were also included as covariates in primary caregiver QALY regression analyses in order to control for imbalances in baseline utility values between arms [29]. Controlling for baseline costs was explored as a scenario analysis [30]. For the cost analysis, generalised linear models using a log link and gamma family form were used to account for the nature of cost data (i.e. mass point at zero, non-negative, skewed) [31]. For the QALY analysis, ordinary least square regressions were used. In line with UK guidelines, costs and QALYs were discounted at 3.5% per annum [25]. Multiple imputation by chained equations with predictive mean matching was used to impute missing cost, outcome and income data; the subsequent analysis of multiple imputed data sets followed Rubin’s rules [32, 33]. Analyses supporting the reliability of the imputation model are provided in Appendix 6 in Supplementary Materials.

Cost-effectiveness results included: adjusted mean cost and QALY estimates, incremental cost-effectiveness ratios (ICERs), incremental net-health benefits (INHB) and probabilities of alternatives being the most costly, effective, and cost-effective strategy. Results were presented for primary caregivers, children, and the dyad (overall) with ICERs representing the cost per additional QALY of E-SEE Steps compared to SAU, and INHB the health gain from E-SEE Steps (in QALYs) above the opportunity cost of its additional expenditure (i.e. the health which would have otherwise been generated elsewhere with those resources) [28]. Monte Carlo simulation using the Cholesky decomposition method of error propagation was employed to capture uncertainty in the estimates [34]. Measures of cost-effectiveness were considered using threshold values used by the UK’s Department for Health [35] (£15,000 per QALY) and National Institute for Health and Care Excellence [25] (£20,000 and £30,000 per QALY). Sensitivity analyses were conducted deterministically. The analysis was prespecified in a health economic analysis plan [19].

## Results

### Patient data and characteristics

Primary caregivers who informed the economic analysis were female, predominately in cohabiting relationships (89%), had undergone further education (68%), and were broadly representative of the UK’s ethnic background. Enrolled children were on average 6-weeks old at baseline and were gender balanced (49% female). Characteristics were comparable between trial arms. Further details on participant characteristics are reported elsewhere [17]. Missing parent and child baseline characteristics, resource use data and HRQoL outcomes were low (< 10%), income data had an elevated degree of missingness (22%).

### Cost analysis

The cost of the E-SEE Steps intervention amounted to £458.50 per primary carer. The main cost drivers were training group leaders (£233.19 per trial participant (50.8%)) and IY-I and IY-T group-session delivery (£196.50 (42.9%)); other programme costs included IY-B book provision (£20 (4.3%)) and contact-related expenses (£8.81 (1.9%)). Average training and group-session costs were highly variable between sites and were contingent on a variety of contextual factors including the number of attendees, catering, venue costs, participant childcare requirements, training expenses and job-profiles of those delivering the course(s). A breakdown of programme costs can be found in Appendix 2–3 in Supplemental Materials.

For primary caregivers and children, average public sector costs were £401 and £161 higher for E-SEE Steps relative to SAU; cost differentials were predominantly driven by intervention costs and community service utilisation, respectively. From a family perspective, average costs were comparable between arms. E-SEE Steps equated to an additional cost of £526 per family when considering both family and public sector perspectives. Table 1 provides a full cost breakdown.

**Table 1:** Adult-related and child-related costs by resource category (pounds sterling, 2018/19)

	**E-SEE Steps**						**SAU**					
	**Adult**		**Child**		**Overall**		**Adult**		**Child**		**Overall**	
	**Mean**	**SD**	**Mean**	**SD**	**Mean**	**SD**	**Mean**	**SD**	**Mean**	**SD**	**Mean**	**SD**
***Public sector perspective***												
Incredible Years	458.50	1021.23	0.00	0.00	458.50	1021.23	0.00	0.00	0.00	0.00	0.00	0.00
Primary care	135.97	173.63	225.12	225.48	361.09	324.01	132.17	163.57	248.75	220.36	380.92	300.64
Secondary care	458.66	1359.10	417.77	830.04	876.43	1664.99	434.64	1602.20	387.14	793.32	821.78	1844.70
Mental health care	205.58	1131.00	23.97	281.52	229.55	1199.39	365.54	1603.58	0.00	0.00	365.54	1603.58
Community based services	323.02	727.41	548.37	803.07	871.39	1268.97	251.34	699.68	413.63	430.47	664.96	915.96
Social services	15.65	104.95	12.77	101.01	28.42	203.23	13.14	93.15	16.58	101.57	29.71	193.43
Total cost	1597.37	2886.56	1228.00	1543.07	2825.37	3709.42	1196.82	3290.44	1066.09	975.85	2262.92	3650.21
***Family perspective***												
Childcare	-	-	1137.19	1054.22	1137.19	1054.22	-	-	1292.30	1435.30	1292.30	1435.30
Absent days from work	517.93	4433.31	-	-	517.93	4433.31	399.61	951.63	-	-	399.61	951.63
Total cost	517.93	4433.31	1137.19	1054.22	1655.12	4621.61	399.61	951.63	1292.30	1435.30	1691.91	2066.91
***Broader perspective***												
Total cost	2115.30	5096.83	2365.19	1965.73	4480.49	5869.73	1596.43	3395.34	2358.40	1674.23	3954.83	4031.70

### Outcome assessment

Compared to baseline, mapped EQ-5D-3L scores for primary caregivers randomised to E-SEE Steps increased at 2-months and 9-months follow-up, before returning to values comparable with baseline figures and SAU at 18-months; scores were highest in E-SEE Steps at all time points. HRQoL scores using EQ-5D-5L preference values found comparable findings, albeit with higher HRQoL values in both arms. Children randomised to E-SEE Steps reported modest increases in child SDQ distress and impairment scores that translated into small decrements in mapped CHU9D HRQoL scores compared to SAU. Full details of imputed HRQoL scores are available in Table 2.

**Table 2:** Imputed adult-related and child-related HRQoL outcomes by treatment group and follow-up period

	**E-SEE Steps**				**Services as usual**				**Difference**			
	**Baseline Mean (SE)**	**FU1 Mean (SE)**	**FU2 Mean (SE)**	**FU3 Mean (SE)**	**Baseline Mean (SE)**	**FU1 Mean (SE)**	**FU2 Mean (SE)**	**FU3 Mean (SE)**	**Baseline Mean (SE)**	**FU1 Mean (SE)**	**FU2 Mean (SE)**	**FU3 Mean (SE)**
***Adult HRQoL***												
EQ-5D-5L	0.93585 (0.005)	0.94729 (0.005)	0.94758 (0.006)	0.93457 (0.006)	0.91457 (0.014)	0.91970 (0.014)	0.91534 (0.018)	0.92878 (0.015)	0.02127 (0.013)	0.02759 (0.013)	0.03224 (0.015)	0.00579 (0.016)
(Mapped) EQ-5D-3L	0.89386 (0.007)	0.91285 (0.007)	0.91859 (0.008)	0.89645 (0.009)	0.86446 (0.019)	0.87181 (0.019)	0.87721 (0.025)	0.89115 (0.020)	0.02940 (0.018)	0.04105 (0.018)	0.04137 (0.020)	0.00530 (0.023)
***Child HRQoL***												
SDQ score	-	-	-	9.67293 (0.262)	-	-	-	9.15094 (0.623)	-	-	-	0.52199 (0.649)
(Mapped) CHU9D	-	-	-	0.85324 (0.002)	-	-	-	0.85933 (0.005)	-	-	-	-0.00609 (0.006)

*FU1* 2-month follow-up, *FU2* 9-month follow-up, *FU3* 18-month follow-up, *SE* Standard error

### Cost-effectiveness

Table 3 reports the mean trial cost, QALY and cost-effectiveness estimates for the dyad and for primary caregivers and children specifically. In primary caregivers, estimated incremental costs and QALYs equalled £446 and 0.034 for E-SEE Steps, respectively, equating to an ICER of £13,011 per QALY compared to SAU. For children, incremental costs were estimated at £178 with a QALY loss of 0.005 compared to SAU, meaning E-SEE steps was dominated by SAU. Overall, E-SEE Steps was estimated to have an incremental cost of £621, incremental QALY gain of 0.031 (QALY gains in primary caregivers surpassing child QALY decrements) and an ICER of approximately £20,062 per QALY compared to SAU. INHB were positive at all cost-effectiveness thresholds considered in primary caregivers but were negative at £15,000 and £20,000 thresholds overall and at all thresholds for children. Probabilities of cost-effectiveness varied widely and were largely contingent on choice of threshold and perspective. Results suggested significant decision uncertainty with 95% credible intervals for INHB crossing zero and cost and QALY intervals overlapping between E-SEE Steps and SAU. All regression results used to inform the cost-effectiveness analysis are reported in Appendix 4 in Supplemental Materials.

**Table 3:** Within-trial cost-effectiveness analysis

Cost-effectiveness	Costs	QALYs	Inc Costs	Inc QALY		Incremental net health benefit (95% CI)		
	(95% CI)	(95% CI)				k = £15,000	k = £20,000	k = £30,000
	[P(most costly)]	[P(most effective)]	(95% CI)	(95% CI)	ICER	[Probability of being cost-effective]		
**Dyad**								
Services as usual	£1,988.61	2.58680				-	-	-
	(146.79, 2615.43)	(2.54927, 2.62129)				-	-	-
	[0.037]	[0.06]				0.640	0.512	0.332
E-SEE Steps	£2,609.46	2.61775	£620.85 (-103.32, 1288.70)	0.03095 (-0.00830, 0.07094)	£20,061.02	-0.01044	-0.00009	0.01025
	(2312.07, 2951.04)	(2.60252, 2.6342)				(-0.07207, 0.05246)	(-0.07207, 0.05246)	(-0.036, 0.0574)
	[0.963]	[0.94]				0.360	0.488	0.668
**Primary carer**								
Services as usual	£942.44	1.31392				-	-	-
	(604.51, 1461.11)	(1.27465, 1.35166)				-	-	-
	[0.052]	[0.044]				0.45	0.316	0.192
E-SEE Steps	£1,388.26	1.34818	£445.82 (-136.90, 890.20)	0.03427 (-0.00643, 0.07679)	£13,010.68	0.004544	0.01197	0.01940
	(1142.31, 1639.19)	(1.33322, 1.36373)				(-0.04636, 0.06414)	(-0.04636, 0.06414)	(-0.0246, 0.06746)
	[0.948]	[0.956]				0.550	0.684	0.808
**Child**								
Services as usual	£1,000.28	1.27420				-	-	-
	(746.07, 1322.25)	(1.26722, 1.28191)				-	-	-
	[0.143]	[0.868]				0.913	0.929	0.940
E-SEE Steps	£1,177.33	1.26957	£177.05 (-175.88 484.85)	-0.00463 (-0.01333, 0.00331)	Dominated	-0.01644	-0.01349	-0.01053
	(1034.65, 1340.39)	(1.26629, 1.27261)				(-0.03847, 0.0087)	(-0.03847, 0.0087)	(-0.03847, 0.0087)
	[0.856]	[0.132]				0.087	0.071	0.060

### Scenario and sensitivity analysis

The dyad trial cost, QALY and ICER estimates for a range of scenario analyses are reported in Table 4. Detailed results for each scenario are provided in Appendix 5 in Supplementary Materials. Controlling for baseline costs increased the incremental cost of E-SEE Steps compared to SAU (from £621 to £815), as SAU accrued higher average costs in the two months prior to enrolment in the trial (i.e. controlling for baseline costs reduced the costs attributable to SAU alone relative to E-SEE Steps). As a result, E-SEE Steps had an ICER of £26,312 per QALY.

**Table 4:** Cost-effectiveness scenario analysis

	Costs	QALYs	ICER		Costs	QALYs	ICER
**Controlling for baseline costs**				**North Yorkshire site costs**			
Services as usual	£1,775.42	2.58680		Services as usual	£1,988.61	2.58680	
E-SEE Steps	£2,589.71	2.61775	£26,312	E-SEE Steps	£2,681.61	2.61775	£22,392
**12 participants per IY-I and IY-T group**				**Blackburn with Darwen site costs**			
Services as usual	£1,988.61	2.58680		Services as usual	£1,988.61	2.58680	
E-SEE Steps	£2,297.85	2.61775	£9,992	E-SEE Steps	£2,595.88	2.61775	£19,622
**EQ5D-5L preference values**				**Suffolk site costs**			
Services as usual	£1,988.61	2.64511		Services as usual	£1,988.61	2.58680	
E-SEE Steps	£2,609.46	2.66805	£27,068	E-SEE Steps	£2,575.83	2.61775	£18,974
**Broader perspective**				**Minimum costs across sites**			
Services as usual	£3,727.13	2.58680		Services as usual	£1,988.61	2.58680	
E-SEE Steps	£4,180.62	2.61775	£14,653	E-SEE Steps	£2,515.23	2.61775	£17,016
**Portsmouth site costs**				**Maximum costs across sites**			
Services as usual	£1,988.61	2.58680		Services as usual	£1966.61	2.58680	
E-SEE Steps	£2,564.65	2.61775	£18,613	E-SEE Steps	£2,727.78	2.61775	£23,820

When using EQ-5D-5L preference weights to measure adult HRQoL, QALY estimates were notably higher compared to base case findings but with E-SEE Steps associated with a smaller QALY gain (0.031 to 0.023) and an ICER of £27,068 per QALY.

Taking a broader costing perspective (public sector and family-related costs) increased overall mean costs in both arms, reduced incremental costs associated with E-SEE Steps compared to base case findings (£621 to £432 – from childcare cost savings) and had an associated ICER of £14,654.

Given the high variability in the attendance of IY-I and IY-T group-sessions during the trial, a best-case scenario assumed all those who attended sessions were in groups at full capacity (12 families). The incremental cost of E-SEE Steps (£312) was approximately half base case values (£621) when run at capacity, with an associated ICER of £9,993 compared to SAU.

E-SEE Steps’ average programme costs were sensitive to changes in number of attendees at group sessions and the number of programmes delivered by each trained practitioner. Group sessions attended by a single primary-caregiver increased intervention costs to an average of £1,423 per family (ICER: £52,110); when attended by two, three or four primary-caregivers average intervention costs reduced to £711 (ICER: £29,137), £475 (ICER: £21,479) and £356 (ICER: £17,650), respectively. Assuming trained group-leaders delivered two, three or four programmes reduced average intervention costs (£486) to £342 (ICER: £17,680), £304 (ICER £16,424) and £284 (ICER: £15,797), respectively. Average intervention costs plateaued to approximately £235 (ICER: £14,300) at 20 programmes delivered per practitioner.

Applying the minimum training expenses and practitioner pay bands observed at trial sites reduced intervention costs by approximately £94 and the associated ICER to £17,017 per QALY. Using the maximum training and staff costs observed across trial sites raised intervention costs by approximately £117 and the associated ICER to £23,820. Results for each specific site fell between the minimum and maximum costing scenarios (Table 4).

## Discussion

Our findings suggest the E-SEE Steps programme offers small health gains to primary caregivers with potentially small detrimental impacts on child outcomes. E-SEE Steps would be borderline cost-effectiveness at UK thresholds over an 18-month time horizon. However, the potential for negative impacts on children and for these to be linked with future detrimental outcomes suggest that the cost-effectiveness of the programme is questionable. Gains in HRQoL were experienced solely by primary caregivers; there was no evidence indicating E-SEE Steps enhanced child social emotional wellbeing nor HRQoL. The average intervention costs equated to £458.50 per dyad, overall incremental costs of E-SEE steps amounted to £621 from a public perspective and had an ICER of £20,062 compared to SAU. Decision uncertainty was large, however scenario analyses found E-SEE Steps remained cost-effective at a £30,000 per QALY threshold for a variety of alternate assumptions. Average programme costs were sensitive to changes in the scale of programme delivery and attendance.

Hurt et al.’s systematic review cited insufficient evidence that early-year programmes are effective at improving childhood development up to 24 months postpartum, a conclusion E-SEE Steps has not altered [36]. However, IY programmes have demonstrated efficacy in older child cohorts in a variety of contexts [13]. The magnitude of costs in our study was significantly below previous findings. This was likely driven by the proportionate delivery of parenting programmes in E-SEE which significantly reduced programme costs compared to such previous assessments of universal provision, even with group sizes in E-SEE significantly below those reported elsewhere [37, 38]. Gardner et al.’s [13] economic analysis of 5 randomised trials reported costs for IY parenting interventions ‘as provided’ ranged between £1,496-£1,792. Our findings offered additional support to evidence from Edwards et al. [37] that non-recurrent fixed costs and course attendance significantly contribute to average programme costs and from O’Neill et al. [38] that staff-expenses constitute the largest cost component of delivering group-sessions and that IY programmes are associated with additional service use compared to control. Despite significantly lower costs, cost-effectiveness results for E-SEE Steps were less favourable than previous analyses of IY programmes [37–39] that compared different configurations of IY serving older children.

The present study had several strengths. Data was compiled from a multi-centre prospective randomised study which achieved high levels of data completion across a number of validated HRQoL instruments and service use questionnaires. The analysis used methods in line with UK guidance for cost-effectiveness analysis, applied cost-effectiveness thresholds applicable for UK decision making, assessed generalisable health outcomes, controlled for adult and child covariables to address potential baseline imbalances, and explored the impacts alternative methodologies, perspectives and structural assumptions had on base case findings.

Nonetheless, the analysis had a number of weaknesses. First, child outcomes were assessed at a single time point using an instrument not validated for study participants (children 20 months of age) [40], meaning the analysis failed to fully account for potential baseline differences or underlying treatment effect dynamics in children and misaligned the study population with those used to validate the SDQ and map preference weights [27, 40]. Second, the generalisability of base case intervention costs may be limited given the trial incurred no venue costs for hosting parenting programmes, fixed costs were only distributed over small samples and broader implementation costs applicable to national roll-outs were not considered (e.g. process factors including administration, procurement and oversight) [41]. In addition, a voucher scheme used to encourage participation during the trial was not costed in the present analysis thereby potentially underestimating total costs in both arms. Third, conclusions on cost-effectiveness relied on modest interim differentials in parental HRQoL informed by a trial that was not powered to detect for differences in adult HRQoL. Fourth, the economic analysis failed to consider impacts outside the dyad despite the trial collecting partial co-parent data. A wider perspective could provide a more comprehensive account of the health, resource, and earning consequences applicable to a household and wider family when undertaking a parental-programme.

Study results were based on intermediary early-year assessments of children and their primary caregivers; however, the longer-term consequences of E-SEE Steps remain unclear. At later stages of development, children may benefit from parental learnings, reductions in caregiver depression scores and uptakes in community services associated with E-SEE Steps. On the other hand, those child decrements reported may persist into the future, and in the longer-term, exceed those temporary gains in HRQoL observed in adults during the trial. Stakeholders are interested in the long-term returns to investment and the overall cost-effectiveness of public health prevention from early-year services. To better inform decision making, programme evaluations would benefit from: 1) long-term follow-up to better assess final child outcomes; 2) generalisable health-related measures that are valid, appropriate, reliable and responsive across different stages of development in young paediatric populations; and 3) the application of life course modelling to best extrapolate study findings [42–44]. Pragmatic observational study designs can help provide longer-term follow-up, larger-scale studies can consider broader operational and contextual factors that may underpin cost-effectiveness, and causal inference methods can provide meaningful estimates of treatment effect in the absence of randomised allocations [41, 45, 46]. Studies that assess longer-term consumption, mortality and HRQoL consequences can benefit from new developments in life-course modelling (e.g. LifeSim) which can extrapolate to ultimate life-time economic, social and health outcomes [44]. Applications of distributional cost-effectiveness and cross‐sectoral frameworks can extend evaluations to consider information about the fairness in the distribution of who gains and who loses from early-year programmes, alternative uses of limited public resources, and best account for the costs and effects that fall on non-health sectors and alternative decision makers [47, 48]

Programmes such as E-SEE Steps with potentially conflicting impacts on adult and child outcomes pose difficult questions for decision making. To what extent should decrements in outcomes for some (e.g. children) be tolerated for benefits to others (e.g. caregivers, siblings, etc.)? Are child-centred interventions ineffective at improving child-outcomes fit for purpose? The E-SEE Steps programme could be considered an effective screening tool for identifying and helping primary caregivers in need of support. Whether the gains in parental HRQoL achieved from doing this constitute a cost-effective allocation of resources is a matter of the perspective, willingness to pay and overarching objectives of decision makers [49].

## Conclusions

In summary, the present study found no evidence that E-SEE Steps improved child outcomes, while conclusions around overall cost-effectiveness relied on moderate intermediary gains in parental HRQoL and intervention costs at approximately £460. Future economic evaluations in parenting interventions can go further by assessing follow-up across more child developmental stages, utilising life-course modelling where possible, and considering broader impacts both within families and across wider society.

## Supplementary Information

**Additional file 1:** **Table 1.** Unit costs. **Table 2.** Average E-SEESteps programme costs (pounds sterling, 2018/19). **Table 3.** Average group-session costs for IY-I and IY-T by resource category (pounds sterling, 2018/19). **Table 4. **Regression analyses. **Table 5. **Scenario analyses. **Table 6. **Inspection of standard errors of multiply imputed data on key dependant variables.

## Data Availability

The data that support the findings of this study are not openly available due to them containing information that could compromise research participant privacy/consent. Requests for participant-level quantitative data and statistical codes should be made to the corresponding author. Data requests will be put forward to members of the original trial management team who will release data on a case-by-case basis.

## Abbreviations

ASQ: Ages & Stages Questionnaires
CHU9D: Child Health Utility Index – 9 Dimension
CI: Credible Interval
EQ-5D: EuroQol—5 Dimension
E-SEE: Enhancing Social-Emotional Health and Wellbeing in the Early Years
HRQoL: Health-Related Quality of Life
ICER: Incremental Cost-Effectiveness Ratio
INHB: Incremental Net Health Benefit
IY: Incredible Years
IY-B: Incredible Years—Book
IY-I: Incredible Years—Infant
IY-T: Incredible Years—Toddler
NCE: National Institute for Health and Care Excellence
NHS: National Health Service
PHQ: Patient Health Questionnaire
PSSRU: Personal social services research unit
QALY: Quality-Adjusted Life Year
RCT: Randomised Control Trial
SAU: Services As Usual
SD: Standard Deviation
SDQ: Strengths and Difficulties Questionnaire
UK: United Kingdom
