# Supplementary material for: The cost-effectiveness of a proportionate parenting programme for primary caregivers and their child: an economic evaluation using evidence from the E-SEE Trial
Source: BMC Health Serv Res. 2022 Jun 23;22:814. doi: 10.1186/s12913-022-08220-x (PMC9219217; doi:10.1186/s12913-022-08220-x)
Supplement: Supplementary file 1 — Additional file 1: Table 1. Unit costs. Table 2. Average E-SEESteps programme costs (pounds sterling, 2018/19). Table 3. Average group-session costs for IY-I and IY-T by resource category (pounds sterling, 2018/19). Table 4. Regression analyses. Table 5. Scenario analyses. Table 6. Inspection of standard errors of multiply imputed data on key dependant variables. [file 12913_2022_8220_MOESM1_ESM.docx]

# Supplementary materials

**TABLE 1:** Unit costs

| **Resource** | **Unit cost** | | | **Source** |
| --- | --- | --- | --- | --- |
|  | **Average** | **Min^*^** | **Max^*^** |  |
| **E-SEE Steps Programme** |  |  |  |  |
| ***IY Infants*** |  |  |  |  |
| **Between-programme fixed costs** |  |  |  |  |
| IY trainer fees & expenses [per site] | £2,842.50 | £1,555.71 | £4,211.18 | Recorded trial cost |
| Venue hire with catering [per site] | £602.35 | £172.80 | £1,330.01 | Recorded trial cost |
| Other equipment/materials [per site] | £100.00 | - | - | Recorded trial cost |
| IY book (for group leaders) [per book] | £20.00 | - | - | Recorded trial cost |
| Workshop manuals [per book] | £20.00 | - | - | Recorded trial cost |
| Programme trainee fees [per participant] | £16.20 | - | - | Recorded trial cost |
| Leader manual and DVD set [per site] | £600.00 | - | - | Recorded trial cost |
| Telephone supervision of team leaders [per site] | £763.81 | £330.00 | £1,294.24 | Recorded trial cost |
| **Within-programme fixed costs** |  |  |  |  |
| IY staff delivery costs [per hour] |  |  |  |  |
| Mental health practitioners | £45.00 | - | - | Personal Social Services Research Unit, 2019 |
| Early years worker / parenting coordinator | £62.53 | - | - | Personal Social Services Research Unit, 2018 |
| Family support worker | £56.84 | - | - | Personal Social Services Research Unit, 2017 |
| Child health worker | £61.50 | - | - | Personal Social Services Research Unit, 2018 |
| Health visitor | £72.56 | - | - | Personal Social Services Research Unit, 2015 |
| Community health nurse | £71.46 | - | - | Personal Social Services Research Unit, 2015 |
| Clinical team lead | £87.00 | - | - | Personal Social Services Research Unit, 2019 |
| Equipment/Materials [up to 12 participants] | £200.00 | - | - | Recorded trial cost |
| Catering [each session for up to 12 participants] | £57.32 | - | - | Recorded trial cost |
| **Programme variable costs** |  |  |  |  |
| IY book [per book] | £20.00 | - | - | Recorded trial cost |
| Child centre/creche venue costs [per hour] | £15.00 | - | - | Recorded trial cost |
| Other forms of contact |  |  |  |  |
| Contact by phone [per call] | £6.00 | - | - | Personal Social Services Research Unit, 2019 |
| Contact by home visit [per visit] | £42.88 | - | - | Personal Social Services Research Unit, 2010 |
| Contact by letter [per letter] | £2.39 | - | - | Gidlow et al (2019) [47] |
| Contact by text [per text] | £0.00 | - | - | Assumed cost-free as texts are already being sent |
| ***IY Toddlers*** |  |  |  |  |
| **Between-programme fixed costs** |  |  |  |  |
| Practitioner training costs |  |  |  |  |
| IY trainer fees and expenses [per site] | £3,014.88 | £2,146.00 | £4,797.50 | Recorded trial cost |
| Venue hire with catering [per site] | £491.19 | £250.00 | £588.00 | Recorded trial cost |
| Other equipment/materials [per site] | £100.00 | - | - | Recorded trial cost |
| IY book [per book] | £16.20 | - | - | Recorded trial cost |
| Workshop manuals [per participant] | £20.00 | - | - | Recorded trial cost |
| Programme trainee fees [per participant] | £16.20 | - | - | Recorded trial cost |
| Leader manual and DVD set [per site] | £750.00 | - | - | Recorded trial cost |
| Telephone supervision of team leaders [per site] | £1,312.38 | £552.50 | £2,687.00 | Recorded trial cost |
| **Within-programme fixed costs** |  |  |  |  |
| IY staff delivery costs (per hour) |  |  |  |  |
| Mental health practitioners | £45.00 | - | - | Personal Social Services Research Unit, 2019 |
| Early years worker / parenting coordinator | £62.53 | - | - | Personal Social Services Research Unit, 2018 |
| Family support worker | £56.84 | - | - | Personal Social Services Research Unit, 2017 |
| Child health worker | £61.50 | - | - | Personal Social Services Research Unit, 2018 |
| Health visitor | £72.56 | - | - | Personal Social Services Research Unit, 2015 |
| Community health nurse | £71.46 | - | - | Personal Social Services Research Unit, 2015 |
| Clinical team lead | £87.00 | - | - | Personal Social Services Research Unit, 2019 |
| Equipment/Materials (for up to 12 participants) | £200.00 | - | - | Recorded trial cost |
| Catering (each session for up to 12 participants) | £57.32 | - | - | Recorded trial cost |
| **Programme variable costs** |  |  |  |  |
| IY book [per book] | £20.00 | - | - | Recorded trial cost |
| Child centre/creche venue costs [per hour] | £15.00 | - | - | Recorded trial cost |
| Other forms of contact |  |  |  |  |
| Contact by phone [per call] | £6.00 | - | - | Personal Social Services Research Unit, 2019 |
| Contact by home visit [per visit] | £42.88 | - | - | Personal Social Services Research Unit, 2010 |
| Contact by letter [per letter] | £2.39 | - | - | Gidlow et al (2019) |
| Contact by text [per text] | £0.00 | - | - | Assumed cost-free as texts are already being sent |
| **Childcare** |  |  |  |  |
| Playgroup [per hour] | £2.50 | - | - | Average childcare costs: Money Advice Service |
| Childminder [per hour] | £4.57 | - | - | Childcare Survey 2019 |
| Family friend [per hour] | £4.57 | - | - | Assumed equal to childminder cost |
| Day nursery [per hour] | £5.16 | - | - | Childcare Survey 2019 |
| **Parenting classes** |  |  |  |  |
| All parenting programmes [per programme] | £1,501.00 | - | - | PSSRU Unit Costs of Health and Social Care 2016 |
| **Health and social care** |  |  |  |  |
| GP surgery visit [per instance] | £33.00 | - | - | Personal Social Services Research Unit, 2019 |
| GP home visit [per instance] | £107.07 | - | - | Personal Social Services Research Unit, 2010 |
| GP doctor phone call [per instance] | £15.32 | - | - | Personal Social Services Research Unit, 2019 |
| GP nurse surgery visit [per instance] | £5.69 | - | - | Personal Social Services Research Unit, 2019 |
| GP nurse home visit [per instance] | £31.44 | - | - | Personal Social Services Research Unit, 2010 |
| GP nurse phone call [per instance] | £6.00 | - | - | Personal Social Services Research Unit, 2019 |
| Health visitor [per instance] | £93.35 | - | - | NHS Reference cost 2017/18 [N03B-N03D] |
| District nurse [per instance] | £39.42 | - | - | NHS Reference cost 2017/18 [N02AF] |
| Other doctor appointment [per instance] | £81.08 | - | - | NHS Reference cost 2017/18 [N29AF] |
| Psychiatrist (adult) [per instance] | £189.72 | - | - | NHS Reference cost 2017/18 [724 - Perinatal Psychiatry] |
| Psychiatrist (child) [per instance] | £283.66 | - | - | NHS Reference cost 2017/18 [MHSTOTHPLC] |
| Psychologist [per instance] | £142.82 | - | - | NHS Reference cost 2017/18 [713 - Psychotherapy] |
| Other counsellor/therapist (adult) [per instance] | £190.57 | - | - | NHS Reference cost 2017/18 [ MHSTOTHPLA] |
| Other counsellor/therapist (child) [per instance] | £283.66 | - | - | NHS Reference cost 2017/18 [ MHSTOTHPLC] |
| Mental health nurse [per instance] | £92.00 | - | - | Personal Social Services Research Unit, 2019 |
| Accident and emergency visit [per instance] | £112.41 | - | - | Personal Social Services Research Unit, 2010 |
| Outpatient appointment [per instance] | £143.73 | - | - | NHS Reference cost 2017/18 [Outpatient - General Surgery] |
| Social worker (office visit) [per instance] | £20.25 | - | - | Personal Social Services Research Unit, 2019 |
| Social worker (home visit) [per instance] | £29.25 | - | - | Personal Social Services Research Unit, 2019 |
| Midwifery [per instance] | £81.61 |  |  | NHS Reference cost 2017/18 [Outpaatient - 560] |
| 111 phone call [per instance] | £13.87 | - | - | Turner J et al (2012) |
| Dentist/Orthodontist [per instance] | £123.38 | - | - | Personal Social Services Research Unit, 2019 |
| Walk-in centre visit [per instance] | £47.15 | - | - | NICE guideline 94 (2018) |
| Dietician [per instance] | £88.15 | - | - | NHS Reference cost 2017/18 [A03] |
| General surgery [per instance] | £143.74 | - | - | NHS Reference cost 2017/18 [A03] |
| Ambulance [per instance] | £190.53 | - | - | National Audit Office 2017 |
| Orthopaedic appointments [per instance] | £127.04 | - | - | NHS Reference cost 2017/18 [Outpatient -110] |
| Smoking cessation support [per instance] | £208.57 | - | - | NHS Reference cost 2017/18 [Day case -DZ58Z] |
| Hearing Assessment [per instance] | £83.77 | - | - | NHS Reference cost 2017/18 [CA37A] |
| **Hospital admissions/procedures** |  |  |  |  |
| Hospital day neonatal [per instance] | £405.00 | - | - | NHS Reference cost 2017/18 [PB06J-PB06M] |
| Hospital day paediatrician [per instance] | £314.97 | - | - | NHS Reference cost 2017/18 [PX57A-PX57C] |
| Birth [per instance] | £2,026.30 | - | - | NHS Reference cost 2017/18 [NZ30A-C] |
| C-section birth [per instance] | £3,809.72 | - | - | NHS Reference cost 2017/18 [NZ50A-C] |
| Miscarriage [per instance] | £1,955.37 | - | - | NHS Reference cost 2017/18 [MB08A] |
| Knee fracture surgery [per instance] | £4,575.33 | - | - | NHS Reference cost 2017/18 [HE21B-HE21BD] |
| General renal disorders [per instance] | £3,919.51 | - | - | NHS Reference cost 2017/18 [LA09L] |
| Cleft surgery [per instance] | £607.04 | - | - | NHS Reference cost 2017/18 [CA66B] |
| Heart surgery [per instance] | £4,336.82 | - | - | NHS Reference cost 2017/18 [ED31A-ED31C] |
| Gall bladder surgery [per instance] | £3,640.81 | - | - | NHS Reference cost 2017/18 [GA10G] |

^*^The minimum/maximum IY costs across participating sites (in cases where costs varied across sites)

**TABLE 2:** Average E-SEE Steps programme costs (pounds sterling, 2018/19)

|  | **Mean** | **SD** |
| --- | --- | --- |
| IY-B book | £20.00 | 0.00 |
| IY-I group costs | £97.08 | 242.75 |
| IY-T group costs | £99.42 | 393.38 |
| IY-I staff training | £158.56 | 379.18 |
| IY-T staff training | £74.63 | 334.26 |
| IY contact | £8.81 | 21.98 |
| Total | £458.50 | 1,021.23 |

**TABLE 3:** Average group-session costs for IY-I and IY-T by resource category (pounds sterling, 2018/19)

| **Average attendee costs** | **IY-I group costs (n=54)** | | **IY-T group costs (n=23)** | |
| --- | --- | --- | --- | --- |
|  | **Mean** | **SD** | **Mean** | **SD** |
| Staff costs | £354.10 | 213.33 | £543.68 | 460.78 |
| Catering | £36.09 | 50.43 | £95.00 | 113.77 |
| Handouts | £38.83 | 17.43 | £42.50 | 28.99 |
| Child centre costs | £83.33 | 185.54 | £550.78 | 378.01 |
| Total average cost | £512.36 | 313.13 | £1,231.96 | 724.56 |

**TABLE 4:** Regression analyses

Base case regression analysis for adult QALYs

| **Base case adult QALYs (EQ5D-3L)** | **Coef.** | **St.Err.** | **t-value** | **p-value** | **[95% Conf** | **Interval]** | | **Sig** |
| --- | --- | --- | --- | --- | --- | --- | --- | --- |
| E-SEE Steps | 0.034 | 0.020 | 1.73 | 0.084 | -0.005 | 0.073 | | * |
| Baseline EQ5D | 0.633 | 0.069 | 9.20 | 0.000 | 0.497 | 0.768 | | *** |
| Baseline PHQ score | -0.013 | 0.002 | -5.31 | 0.000 | -0.018 | -0.008 | | *** |
| Baseline ASQ score | 0.000 | 0.001 | -0.79 | 0.430 | -0.001 | 0.001 | |  |
| *Child age* |  |  |  |  |  |  | |  |
| 1 | -0.031 | 0.020 | -1.58 | 0.115 | -0.070 | 0.008 | |  |
| 2 | -0.048 | 0.024 | -2.04 | 0.041 | -0.095 | -0.002 | | ** |
| Parent age | 0.001 | 0.002 | 0.67 | 0.504 | -0.002 | 0.004 | |  |
| Child Gender (boy) | -0.013 | 0.015 | -0.90 | 0.369 | -0.042 | 0.016 | |  |
| *Highest qualification* |  |  |  |  |  |  | |  |
| Overseas qualification | 0.044 | 0.043 | 1.02 | 0.309 | -0.041 | 0.128 | |  |
| GCSE<5 | -0.045 | 0.049 | -0.91 | 0.368 | -0.144 | 0.055 | |  |
| GCSE≥5 | -0.009 | 0.034 | -0.28 | 0.780 | -0.076 | 0.057 | |  |
| A-levels | -0.007 | 0.032 | -0.21 | 0.833 | -0.069 | 0.056 | |  |
| HE-diploma | -0.025 | 0.028 | -0.88 | 0.378 | -0.080 | 0.031 | |  |
| BA/Bsc | 0.003 | 0.022 | 0.13 | 0.900 | -0.040 | 0.045 | |  |
| MA/Msc | 0.002 | 0.029 | 0.08 | 0.937 | -0.055 | 0.060 | |  |
| *Ethnicity* |  |  |  |  |  |  | |  |
| Multiple ethnicities | 0.079 | 0.047 | 1.69 | 0.091 | -0.013 | 0.171 | | * |
| Asian/British-Asian | 0.026 | 0.022 | 1.17 | 0.243 | -0.017 | 0.069 | |  |
| Black/Black-British | 0.056 | 0.049 | 1.15 | 0.252 | -0.040 | 0.153 | |  |
| Other ethnic group | 0.000 | 0.131 | -0.00 | 0.997 | -0.258 | 0.257 | |  |
| *Relationship status* |  |  |  |  |  |  | |  |
| Cohabitation | 0.028 | 0.019 | 1.47 | 0.142 | -0.010 | 0.066 | |  |
| Couple | -0.108 | 0.057 | -1.91 | 0.056 | -0.219 | 0.003 | | * |
| Apart | -0.036 | 0.037 | -0.97 | 0.336 | -0.111 | 0.039 | |  |
| Constant | 0.795 | 0.083 | 9.59 | 0.000 | 0.633 | 0.958 | | *** |
| ****** p<0.01, ** p<0.05, * p<0.1*** | | | | | | |  |  |

Base case regression analysis for child QALYs

| **Base case child QALYs (CHU-9D)** | **Coef.** | **St.Err.** | **t-value** | **p-value** | **[95% Conf** | **Inter-val]** | **Sig** |
| --- | --- | --- | --- | --- | --- | --- | --- |
| E-SEE Steps | -0.004 | 0.004 | -1.06 | 0.289 | -0.013 | 0.004 |  |
| Baseline ASQ score | 0.000 | 0.000 | -2.20 | 0.028 | 0.000 | 0.000 | ** |
| Baseline PHQ score | -0.001 | 0.000 | -1.45 | 0.148 | -0.002 | 0.000 |  |
| Child age |  |  |  |  |  |  |  |
| 1 | 0.002 | 0.004 | 0.39 | 0.695 | -0.007 | 0.010 |  |
| 2 | -0.006 | 0.005 | -1.23 | 0.218 | -0.016 | 0.004 |  |
| Parent age | 0.000 | 0.000 | 0.61 | 0.541 | 0.000 | 0.001 |  |
| Child Gender (boy) | -0.006 | 0.003 | -1.82 | 0.069 | -0.012 | 0.000 | * |
| Highest qualification |  |  |  |  |  |  |  |
| Overseas qualification | -0.004 | 0.010 | -0.45 | 0.654 | -0.024 | 0.015 |  |
| GCSE<5 | -0.008 | 0.009 | -0.89 | 0.372 | -0.026 | 0.010 |  |
| GCSE≥5 | 0.007 | 0.007 | 0.95 | 0.345 | -0.008 | 0.022 |  |
| A-levels | 0.003 | 0.007 | 0.48 | 0.635 | -0.011 | 0.018 |  |
| HE-diploma | 0.002 | 0.006 | 0.36 | 0.715 | -0.010 | 0.015 |  |
| BA/Bsc | 0.010 | 0.005 | 2.15 | 0.032 | 0.001 | 0.019 | ** |
| MA/Msc | 0.013 | 0.006 | 2.11 | 0.035 | 0.001 | 0.025 | ** |
| Ethnicity |  |  |  |  |  |  |  |
| Multiple ethnicities | 0.006 | 0.010 | 0.64 | 0.523 | -0.013 | 0.025 |  |
| Asian/British-Asian | 0.000 | 0.005 | -0.07 | 0.948 | -0.010 | 0.009 |  |
| Black/Black-British | -0.001 | 0.012 | -0.09 | 0.925 | -0.025 | 0.023 |  |
| Other ethnic group | 0.034 | 0.027 | 1.24 | 0.217 | -0.020 | 0.087 |  |
| Relationship status |  |  |  |  |  |  |  |
| Cohabitation | 0.004 | 0.004 | 0.89 | 0.374 | -0.004 | 0.011 |  |
| Couple | 0.002 | 0.013 | 0.13 | 0.894 | -0.023 | 0.026 |  |
| Apart | 0.006 | 0.007 | 0.87 | 0.385 | -0.008 | 0.021 |  |
| Constant | 1.272 | 0.012 | 103.21 | 0.000 | 1.247 | 1.296 | *** |
| ***** p<0.01, ** p<0.05, * p<0.1** | | | | | | |  |

Base case regression analysis for adult costs

| **Base case adult (log) costs** | **Coef.** | | | **St.Err.** | **t-value** | | **p-value** | | **[95% Conf** | | | **Interval]** | **Sig** | | |
| --- | --- | --- | --- | --- | --- | --- | --- | --- | --- | --- | --- | --- | --- | --- | --- |
| E-SEE Steps | | 0.406 | 0.252 | | | 1.61 | | 0.108 | | -0.090 | 0.901 | | |  |  |
| Child age | |  |  | | |  | |  | |  |  | | |  |  |
| 1 | | -0.222 | 0.227 | | | -0.98 | | 0.327 | | -0.666 | 0.222 | | |  |  |
| 2 | | -0.266 | 0.282 | | | -0.94 | | 0.346 | | -0.822 | 0.289 | | |  |  |
| Parent age | | -0.023 | 0.018 | | | -1.28 | | 0.200 | | -0.058 | 0.012 | | |  |  |
| Child Gender (boy) | | 0.140 | 0.170 | | | 0.82 | | 0.410 | | -0.193 | 0.473 | | |  |  |
| Highest qualification | |  |  | | |  | |  | |  |  | | |  |  |
| Overseas qualification | | -1.495 | 0.640 | | | -2.34 | | 0.030 | | -2.832 | -0.159 | | | ** |  |
| GCSE<5 | | 0.927 | 0.486 | | | 1.91 | | 0.057 | | -0.027 | 1.881 | | | * |  |
| GCSE≥5 | | 0.146 | 0.464 | | | 0.31 | | 0.754 | | -0.782 | 1.074 | | |  |  |
| A-levels | | -0.042 | 0.360 | | | -0.12 | | 0.908 | | -0.750 | 0.667 | | |  |  |
| HE-diploma | | -0.122 | 0.318 | | | -0.39 | | 0.700 | | -0.745 | 0.501 | | |  |  |
| BA/Bsc | | -0.453 | 0.262 | | | -1.73 | | 0.085 | | -0.969 | 0.063 | | | * |  |
| MA/Msc | | -0.202 | 0.349 | | | -0.58 | | 0.564 | | -0.892 | 0.488 | | |  |  |
| Ethnicity | |  |  | | |  | |  | |  |  | | |  |  |
| Multiple ethnicities | | -0.199 | 0.529 | | | -0.38 | | 0.707 | | -1.237 | 0.839 | | |  |  |
| Asian/British-Asian | | -0.203 | 0.261 | | | -0.78 | | 0.437 | | -0.717 | 0.311 | | |  |  |
| Black/Black-British | | -0.516 | 0.551 | | | -0.94 | | 0.349 | | -1.596 | 0.564 | | |  |  |
| Other ethnic group | | -1.355 | 1.454 | | | -0.93 | | 0.352 | | -4.205 | 1.495 | | |  |  |
| Relationship status | |  |  | | |  | |  | |  |  | | |  |  |
| Cohabitation | | -0.035 | 0.259 | | | -0.14 | | 0.894 | | -0.560 | 0.490 | | |  |  |
| Couple | | 0.651 | 0.634 | | | 1.03 | | 0.305 | | -0.593 | 1.895 | | |  |  |
| Apart | | 0.537 | 0.471 | | | 1.14 | | 0.265 | | -0.430 | 1.503 | | |  |  |
| Constant | | 7.888 | 0.669 | | | 11.79 | | 0.000 | | 6.574 | 9.202 | | | *** |  |
| ***** p<0.01, ** p<0.05, * p<0.1** | | | | | | | | | | | | | |  |  |

Base case regression analysis for child costs

| **Base case child (log) costs** | **Coef.** | **St.Err.** | **t-value** | **p-value** | **[95% Conf** | **Interval]** | **Sig** |
| --- | --- | --- | --- | --- | --- | --- | --- |
| E-SEE Steps | 0.173 | 0.157 | 1.10 | 0.271 | -0.135 | 0.482 |  |
| Child age |  |  |  |  |  |  |  |
| 1 | -0.094 | 0.158 | -0.59 | 0.553 | -0.403 | 0.216 |  |
| 2 | -0.208 | 0.191 | -1.09 | 0.274 | -0.582 | 0.165 |  |
| Parent age | -0.028 | 0.013 | -2.12 | 0.035 | -0.055 | -0.002 | ** |
| Child Gender (boy) | 0.120 | 0.118 | 1.01 | 0.311 | -0.112 | 0.351 |  |
| Highest qualification |  |  |  |  |  |  |  |
| Overseas qualification | -0.716 | 0.343 | -2.09 | 0.037 | -1.389 | -0.043 |  |
| GCSE<5 | 0.129 | 0.369 | 0.35 | 0.727 | -0.606 | 0.865 | ** |
| GCSE≥5 | 0.196 | 0.294 | 0.67 | 0.508 | -0.389 | 0.780 |  |
| A-levels | 0.130 | 0.253 | 0.51 | 0.608 | -0.368 | 0.627 |  |
| HE-diploma | 0.068 | 0.226 | 0.30 | 0.765 | -0.377 | 0.512 |  |
| BA/Bsc | 0.149 | 0.182 | 0.82 | 0.413 | -0.209 | 0.508 |  |
| MA/Msc | 0.098 | 0.240 | 0.41 | 0.683 | -0.375 | 0.571 |  |
| Ethnicity |  |  |  |  |  |  |  |
| Multiple ethnicities | -0.271 | 0.366 | -0.74 | 0.458 | -0.988 | 0.445 |  |
| Asian/British-Asian | -0.170 | 0.181 | -0.94 | 0.347 | -0.525 | 0.184 |  |
| Black/Black-British | -0.531 | 0.389 | -1.36 | 0.173 | -1.295 | 0.233 |  |
| Other ethnic group | 0.291 | 1.029 | 0.28 | 0.778 | -1.726 | 2.308 |  |
| Relationship status |  |  |  |  |  |  |  |
| Cohabitation | 0.157 | 0.161 | 0.98 | 0.331 | -0.162 | 0.476 |  |
| Couple | 0.641 | 0.430 | 1.49 | 0.136 | -0.202 | 1.484 |  |
| Apart | 0.251 | 0.354 | 0.71 | 0.487 | -0.488 | 0.990 |  |
| Constant | 7.717 | 0.454 | 17.00 | 0.000 | 6.826 | 8.608 | *** |
|  | | | | | | |  |

***** p<0.01, ** p<0.05, * p<0.1**

Base case regression analysis for overall costs

| **Base case overall (log) costs** | **Coef.** | **St.Err.** | **t-value** | **p-value** | **[95% Conf** | **Interval]** | **Sig** |
| --- | --- | --- | --- | --- | --- | --- | --- |
| E-SEE Steps | 0.285 | 0.163 | 1.75 | 0.081 | -0.035 | 0.605 | * |
| *Child age* |  |  |  |  |  |  |  |
| 1 | -0.189 | 0.153 | -1.24 | 0.217 | -0.489 | 0.111 |  |
| 2 | -0.252 | 0.188 | -1.34 | 0.181 | -0.620 | 0.117 |  |
| Parent age | -0.025 | 0.013 | -2.02 | 0.044 | -0.050 | -0.001 | ** |
| Child Gender (boy) | 0.126 | 0.115 | 1.09 | 0.275 | -0.100 | 0.352 |  |
| *Highest qualification* |  |  |  |  |  |  |  |
| Overseas qualification | -1.088 | 0.398 | -2.73 | 0.010 | -1.895 | -0.281 | ** |
| GCSE<5 | 0.692 | 0.342 | 2.02 | 0.044 | 0.019 | 1.365 | ** |
| GCSE≥5 | 0.142 | 0.298 | 0.47 | 0.636 | -0.454 | 0.737 |  |
| A-levels | 0.048 | 0.249 | 0.19 | 0.846 | -0.442 | 0.539 |  |
| HE-diploma | -0.021 | 0.215 | -0.10 | 0.922 | -0.442 | 0.399 |  |
| BA/Bsc | -0.152 | 0.175 | -0.87 | 0.386 | -0.495 | 0.192 |  |
| MA/Msc | -0.059 | 0.240 | -0.25 | 0.805 | -0.534 | 0.415 |  |
| *Ethnicity* |  |  |  |  |  |  |  |
| Multiple ethnicities | -0.275 | 0.360 | -0.76 | 0.445 | -0.979 | 0.430 |  |
| Asian/British-Asian | -0.177 | 0.183 | -0.97 | 0.335 | -0.537 | 0.184 |  |
| Black/Black-British | -0.474 | 0.370 | -1.28 | 0.200 | -1.200 | 0.251 |  |
| Other ethnic group | -0.090 | 1.006 | -0.09 | 0.929 | -2.062 | 1.882 |  |
| *Relationship status* |  |  |  |  |  |  |  |
| Cohabitation | 0.072 | 0.168 | 0.43 | 0.669 | -0.264 | 0.409 |  |
| Couple | 0.621 | 0.428 | 1.45 | 0.146 | -0.217 | 1.460 |  |
| Apart | 0.440 | 0.342 | 1.29 | 0.213 | -0.273 | 1.153 |  |
| Constant | 8.517 | 0.451 | 18.87 | 0.000 | 7.629 | 9.405 | *** |
|  | | | | | | | |
| ****** p<0.01, ** p<0.05, * p<0.1*** | | | | | | |  |

**TABLE 5: Scenario analyses**

|  |  | **Costs** | **QALYs** | **ICER** | **Incremental net health benefit (95% CI)** | | |
| --- | --- | --- | --- | --- | --- | --- | --- |
|  |  | **(95% CI)** | **(95% CI)** |  | **k=£15,000** | **k=£20,000** | **k=£30,000** |
|  |  | **[P(most costly)]** | **[P(most effective)]** |  | **[Probability of being cost-effective]** | | |
| **EQ-5D-5L** | | | | | | | |
| **Services as usual** | | £1,988.61 | 2.64511 |  | - | - | - |
|  |  | (1465.79, 2615.43) | (2.61663, 2.67177) |  | - | - | - |
|  |  | [0.037] | [0.06] |  | 0.763 | 0.658 | 0.461 |
| **Incredible Years** | | £2,609.46 | 2.66805 | £27,067.81 | -0.01845 | -0.00915 | 0.00224 |
|  |  | (2312.07, 2951.04) | (2.65684, 2.67932) |  | (-0.07096, 0.03719) | (-0.07096, 0.03719) | (-0.03315, 0.03823) |
|  |  | [0.963] | [0.940] |  | 0.237 | 0.342 | 0.539 |
| **Controlling for baseline costs** | | | | | | | |
| **Services as usual** | | £1,775.42 | 2.58680 |  | - | - | - |
|  |  | (1347.82, 2295.03) | (2.54927, 2.62129) |  | - | - | - |
|  |  | [0.003] | [0.06] |  | 0.796 | 0.65 | 0.43 |
| **Incredible Years** | | £2,589.71 | 2.61775 | £26,311.51 | -0.02334 | -0.00977 | 0.00381 |
|  |  | (2287.81, 2923.25) | (2.60252, 2.6342) |  | (-0.07642, 0.0346) | (-0.07642, 0.0346) | (-0.04136, 0.05025) |
|  |  | [0.997] | [0.940] |  | 0.204 | 0.350 | 0.570 |
| **Broader perspective** | | | | | | | |
| **Services as usual** | | £3,727.13 | 2.58680 |  | - | - | - |
|  |  | (2967.99, 4712.34) | (2.54927, 2.62129) |  | - | - | - |
|  |  | [0.150] | [0.060] |  | 0.504 | 0.389 | 0.27 |
| **Incredible Years** | | £4,180.62 | 2.61775 | £14,653.37 | 0.00072 | 0.00827 | 0.01583 |
|  |  | (3783.65, 4605.28) | (2.60252, 2.6342) |  | (-0.06576, 0.07446) | (-0.06576, 0.07446) | (-0.03137, 0.06575) |
|  |  | [0.850] | [0.940] |  | 0.496 | 0.611 | 0.730 |
| **12 per IY group** | | | | | | | |
| **Services as usual** | | £1,988.61 | 2.58680 |  | - | - | - |
|  |  | (1465.79, 2615.43) | (2.54927, 2.62129) |  | - | - | - |
|  |  | [0.175] | [0.060] |  | 0.378 | 0.282 | 0.181 |
| **Incredible Years** | | £2,297.85 | 2.61775 | £9,992.12 | 0.01033 | 0.01356 | 0.02064 |
|  |  | (2041, 2577.04) | (2.60252, 2.6342) |  | (-0.04602, 0.06927) | (-0.04602, 0.06927) | (-0.02363, 0.06488) |
|  |  | [0.825] | [0.940] |  | 0.622 | 0.718 | 0.819 |
| **Minimum site costs** | | | | | | | |
| **Services as usual** | | £1,988.61 | 2.58680 |  | - | - | - |
|  |  | (1465.79, 2615.43) | (2.54927, 2.62129) |  | - | - | - |
|  |  | [0.056] | [0.060] |  | 0.559 | 0.435 | 0.285 |
| **Incredible Years** | | £2,515.23 | 2.61775 | £17,016.26 | -0.00416 | 0.00342 | 0.01339 |
|  |  | (2241.17, 2821.85) | (2.60252, 2.6342) |  | (-0.06113, 0.05469) | (-0.06113, 0.05469) | (-0.03113, 0.06006) |
|  |  | [0.944] | [0.940] |  | 0.441 | 0.565 | 0.715 |
| **Maximum site costs** | | | | | | | |
| **Services as usual** | | £1,988.61 | 2.58680 |  | - | - | - |
|  |  | (1465.79, 2615.43) | (2.54927, 2.62129) |  | - | - | - |
|  |  | [0.018] | [0.060] |  | 0.738 | 0.61 | 0.4 |
| **Incredible Years** | | £2,725.78 | 2.61775 | £23,819.83 | -0.01820 | -0.00562 | 0.00638 |
|  |  | (2411.59, 3069.14) | (2.60252, 2.6342) |  | (-0.0761, 0.04239) | (-0.0761, 0.04239) | (-0.03927, 0.05133) |
|  |  | [0.982] | [0.940] |  | 0.262 | 0.390 | 0.600 |

**TABLE 6:** Inspection of standard errors of multiply imputed data on key dependant variables

|  | **Base case imputations** | | **How_many_imputation recommended imputations^1^** | |
| --- | --- | --- | --- | --- |
| **Variable** | Number | Standard error | Recommended number | Standard error |
| **QALYs** |  |  |  |  |
| Discounted adult QALYs (EQ5D-5L) | 5 |  | 4 | Recommend<Base case |
| Discounted adult QALYs (EQ5D-3L) | 5 |  | 4 | Recommend<Base case |
| **Costs** |  |  |  |  |
| Total costs | 5 | 194 | 13 | 199 |
| Adult total costs | 5 | 160 | 7 | 161 |
| Child total costs | 5 | 79 | 22 | 76 |

^1^ von Hippel PT. How Many Imputations Do You Need? A Two-stage Calculation Using a Quadratic Rule. Sociological Methods & Research. 2020;49(3):699-718. doi:10.1177/0049124117747303

Pattern of missing data

Black shading represents missing data for one or more individuals (arrayed along the horizontal axis) on a particular variable (arrayed along the vertical axis); grey shading

represents observed data

Logistic regression – predictors of missing data for key dependant variables

|  | Missing total public costs | | | | | | | Missing EQ-5D | | | | | | |  |  |
| --- | --- | --- | --- | --- | --- | --- | --- | --- | --- | --- | --- | --- | --- | --- | --- | --- |
|  | Month 2 | | Month 9 | | | Month 18 | | Month 2 | | Month 9 | | | Month 18 | | |  |
|  | β | SE | β | SE | β | | SE | β | SE | β | SE | β | | SE | | |
| E-SEE Steps | 1.232 | 1.066 | 1.503 | 1.113 | 0.043 | | 0.667 | 1.232 | 1.066 | 1.503 | 1.113 | 0.043 | | 0.667 | | |
| Baseline family costs | 0.000 | 0.000 | 0.000 | 0.000 | 0.000 | | 0.000 | 0.000 | 0.000 | 0.000 | 0.000 | 0.000 | | 0.000 | | |
| Baseline ASQ score | -0.029 | 0.022 | -0.049** | 0.024 | -0.042* | | 0.022 | -0.029 | 0.022 | -0.049* | 0.024 | -0.042* | | 0.022 | | |
| Baseline PHQ score | -0.021 | 0.089 | 0.004 | 0.079 | 0.050 | | 0.070 | -0.021 | 0.089 | 0.004 | 0.079 | 0.050 | | 0.070 | | |
| Baseline EQ-5D | -2.505 | 2.392 | -3.872* | 2.350 | 0.303 | | 2.258 | -2.505 | 2.392 | -3.872 | 2.350 | 0.303 | | 2.258 | | |
| Child age |  |  |  |  |  | |  |  |  |  |  |  | |  | | |
| 1 | -0.178 | 0.714 | -0.591 | 0.661 | -0.396 | | 0.638 | -0.178 | 0.714 | -0.591 | 0.661 | -0.396 | | 0.638 | | |
| 2 | 0.539 | 0.797 | -0.098 | 0.794 | 0.009 | | 0.735 | 0.539 | 0.797 | -0.098 | 0.794 | 0.009 | | 0.735 | | |
| Parent age | -0.034 | 0.051 | -0.026 | 0.052 | -0.101 | | 0.049 | -0.034 | 0.051 | -0.026 | 0.052 | -0.101* | | 0.049 | | |
| Child Gender (boy) | 0.309 | 0.525 | 0.320 | 0.530 | 0.059 | | 0.485 | 0.309 | 0.525 | 0.320 | 0.530 | 0.059 | | 0.485 | | |
| Constant | -0.082 | 2.864 | 0.633 | 2.910 | 0.630 | | 2.688 | -0.082 | 2.864 | 0.633 | 2.910 | 0.630 | | 2.688 | | |

*** p<0.01, ** p<0.05, * p<0.1

We believe the standard errors using 5 imputations were generally sufficient compared to those when using the recommended imputations for each variable using the recommended number for each variable^1^. Predictors of missingness were generally inconclusive, missingness was relatively small which reduced power. Minor evidence found that parents with higher HRQoL/child-related asq_score at baseline were less likely to fail to complete cost/eq-5d forms.
